# Synthetic γ‐AApeptides as Targeting Ligands for Immunomagnetic separation of Alzheimer’s disease (AD) Biomarkers

**DOI:** 10.1002/alz.093525

**Published:** 2025-01-09

**Authors:** Yuancheng Li, Hui Mao, Jianfeng Cai, Hedi Ma, Jonathan Padelford

**Affiliations:** ^1^ 5M Biomed, LLC, Altanta, GA USA; ^2^ Emory University School of Medicine, Atlanta, GA USA; ^3^ University of South Florida, Tampa, FL USA; ^4^ 5M Biomed, LLC, Atlanta, GA USA

## Abstract

**Background:**

Liquid biopsy for Alzheimer’s disease (AD) biomarkers in cerebral spinal fluid (CSF) or blood samples is becoming a significant sector in the management of AD and AD related dementia (ADRD). Whereas different types of molecules, such as small molecules, peptides, and antibodies, have been used as ligands for targeting AD biomarkers/pathologies such as amyloid beta peptides (Aβs), current liquid biopsy approaches mostly use antibodies. However, the activity and affinity of the antibodies can vary depending on the source and preparation as well as the stability in storage. This study reports the application of Aβ40‐specific synthetic γ‐AApeptides, consisting of N‐acylated‐N‐aminoethyl amino acid units, as targeting ligands for immunomagnetic separation of Aβ40.

**Method:**

A γ‐AApeptides library was developed using combinatorial chemistry, and screened for Aβ40‐targeting candidates following published methods. Chemical structures of γ‐AApeptides were solved by nuclear magnetic resonance (NMR) spectroscopy and tandem mass spectrometry (MS/MS). Conjugation of γ‐AApeptides with ultra‐magnetic iron oxide nanorod Magfiniti^®^ was performed using the terminal thiol groups on γ‐AApeptides and amine groups on Magfiniti^®^, facilitated by a sulfosuccinimidyl 4‐(N‐maleimidomethyl)cyclohexane‐1‐carboxylate (sulfo‐SMCC) linker. Conjugation was validated by the change of hydrodynamic size and zeta‐potential of Magfiniti^®^. Human serum spiked with fluorescence‐labeled Aβ40 (100 to 2000 pg/mL) was used to test the detection sensitivity of γ‐AApeptide‐Magfiniti^®^, with Aβ40‐targeting 6E10‐antibody‐conjugated Magfiniti^®^ used as control.

**Result:**

We found three structures of γ‐AApeptides specific for Aβ40, namely A‐28, C‐231, and F‐12, all of which contained a backbone of four monomeric γ‐peptide units but different N‐acylated‐N‐aminoethyl sidechains. With similar chemical structures, successful conjugation of each γ‐AApeptides on Magfiniti^®^ was confirmed by the increased hydrodynamic sizes from 58.8±8.0 nm to 91.3±11.9 nm, along with the decrease of zeta potentials from 25.4±7.7 mV to 14.1±5.6 mV. The detection sensitivity for 100 pg/mL Aβ40 by each γ‐AApeptide‐Magfiniti^®^ was 96.1±2.7% (F‐12), 92.2±4.4% (C‐231), and 78.8±6.9% (A‐28), whereas 6E10‐Magfiniti^®^ captured 98.4±2.1% of Aβ40 under same condition.

**Conclusion:**

We synthesized and identified three Aβ40‐specific γ‐AApeptide that can be used as targeting ligands for immunomagnetic separation of AD biomarkers. The γ‐AApeptide F‐12 achieved a similar sensitivity to that of Aβ40‐targeted antibody.

